# Protocol for the “magnitude of cigarette substitution after initiation of e-cigarettes and its impact on biomarkers of exposure and potential harm in dual users” (MAGNIFICAT) study

**DOI:** 10.3389/fpubh.2024.1348389

**Published:** 2024-03-21

**Authors:** Riccardo Polosa, Nikola Pluym, Max Scherer, Jonathan Belsey, Christopher Russell, Pasquale Caponnetto, Jakub Weglarz, Davide Campagna

**Affiliations:** ^1^Department of Clinical and Experimental Medicine, University of Catania, Catania, Italy; ^2^Centre for the Prevention and Treatment of Tobacco Addiction (CPCT), University Teaching Hospital "Policlinico-S.Marco", University of Catania, Catania, Italy; ^3^Center of Excellence for the Acceleration of Harm Reduction (CoEHAR), University of Catania, Catania, Italy; ^4^ABF Analytisch-Biologisches Forschungslabor GmbH, Planegg, Germany; ^5^JB Medical Ltd, London, United Kingdom; ^6^Russell Burnett Research & Consultancy Ltd, Glasgow, United Kingdom; ^7^Department of Science of Education, Section of Psychology, University of Catania, Catania, Italy; ^8^ECLAT Srl, Spin-off of the University of Catania, Catania, Italy; ^9^UOC MCAU, University Teaching Hospital "Policlinico-S.Marco", University of Catania, Catania, Italy

**Keywords:** cigarette and e-cigarette smoking, smoking addiction, health, impact of smoking, biomarkers of exposure, dual use smokers, new heated tobacco products, impact of smoking on respiratory system

## Abstract

**Introduction:**

Many smokers who use e-cigarettes (ECs) to quit continue smoking alongside vaping. The impact on health among individuals who simultaneously smoke conventional cigarettes (CCs) and use ECs remains unclear. The varying patterns of dual use present differing levels of overall toxin exposure and relative risks concerning smoking-related diseases. Understanding these complexities is vital to assessing the implications for human health.

**Objective:**

Herein we describe a protocol designed to analyze the impact of different level of substituting CCs with ECs on exposure to toxicants. We’ll use biomarkers to measure this exposure and assess harm reduction in dual users through clinical endpoints, harm-related biomarkers, and behavioral correlations. We expect to observe progressive changes with varying patterns of dual use.

**Methods and analyses:**

For this purpose, we planned to recruit a group of 250 smokers who will be asked to reduce their CC consumption by adopting ECs (intervention group). A separate group of 50 smokers will continue to smoke CC (reference group). Study groups will be followed up for 6 months during which biospecimens will be collected for biomarker analyses, and clinical endpoints will be assessed. The trial is structured to characterize subjects’ usage patterns over time using robust biomarkers of exposure and a standardized mobile phone application to facilitate the precise categorization of dual users along the risk continuum based on their usage behaviors. Subject recruitment will start in February 2024 and enrolment is expected to be completed by August 2024. Results will be reported early in 2025. Study findings may provide valuable insights into health benefits or risks associated with varying patterns of dual use.

**Ethics and dissemination:**

The study protocol and informed consent forms will be approved by the local Ethical Review Boards. Study results will be disseminated through articles published in reputable, peer-reviewed, open access, scientific journals, presentations at conferences, and the University website.

## 1 Introduction

E-cigarettes (ECs), and heated tobacco products (HTPs) have evolved as popular, yet controversial, combustible cigarette (CC) substitutes among smokers worldwide (1–5).

Although ECs offer substantial reduction in exposure to toxic chemicals compared to CC (6–9), and are useful for harm reduction from cigarette smoke and for smoking cessation (10–13), considerable controversy regarding their risk and potential impact on public health continues to surround the use of these products (14–17).

While completely quitting tobacco smoking by switching to exclusive EC use is associated with a significant reduction in the exposure to harmful constituents and measurable health improvement (18–22), many users continue to smoke while using them. In the United States, the number of adults who currently both smoke cigarettes and use ECs (i.e., dual users) is estimated at 40–60% (23, 24). In Great Britain, 35% of regular vapers also smoke (25). In Japan (by far the largest HTP market in the world), the number of individuals who smoke cigarettes and use HTPs is estimated at about 20% (26, 27).

Given the prevalence of dual use, it is important to understand more about their exposure profile and their risk related to the concomitant smoking of CC and EC use compared to smoking alone. The extent to which smoking-related harm is reduced when EC are used concurrently with CC is less clear (18–22). Although it is a common misconception to consider dual use as a homogenous dichotomous group, dual users are highly heterogeneous in their use of these products (28). Different dual use patterns exist (e.g., strong switchers vs. light switchers) and are likely to have a different impact on overall toxin exposure and relative risk of developing smoking-related disease.

Short-term studies have indicated potential harm reduction for dual cigarette-EC users through reductions in biomarkers of exposure (29–31). However, these studies have limitations, such as the small number of biomarkers investigated, and the short-term setting. Furthermore, these reports rely solely on self-reports without further verification with suitable biomarkers, which could lead to a misinterpretation on the extent of harm reduction. Remarkably these studies do not provide information about the impact of different dual use patterns on biomarkers of exposure and health effect indicators.

This study seeks to bridge these gaps by conducting a systematic assessment of various dual-use patterns through a longitudinal clinical study. Through the compilation of a comprehensive dataset featuring well-defined dual-use patterns, this study aims to facilitate a robust risk assessment of vaping products, while also delving into the health risks and benefits linked to dual use. By stratifying dual users into sub-groups according to their usage patterns - encompassing strong, moderate, and limited substitution of CC with ECs - this research will provide valuable insights into the degree of harm reduction that can be achieved when dual using these products.

This switching study will be conducted as a longitudinal cohort investigation of 300 individuals who smoke, comprising those inclined to transition to ECs as substitutes for CC, alongside a control group of 50 persistent smokers. Utilizing consumption diaries, tracker apps, and pertinent biomarkers indicative of compliance, the extent of reduction in CC consumption will be meticulously evaluated. Dual users will be categorized into sub-groups predicated on the degree of reduction in CC consumption.

This research design is poised to facilitate an evaluation of the decrease in exposure and associated risks for smokers who curtail their CC consumption by switching to EC use. The study’s endpoints encompass a spectrum of measurements, including biomarkers signaling exposure and potential harm, cardiovascular and respiratory functionalities, in addition to self-reported outcomes by study participants.

An essential facet of this investigation entails testing a hypothesis pertaining to a risk continuum, which is principally tied to the level of the risk/harm mitigation attributed to the number of cigarettes smoked per day (CPD) after switching to EC use. Through correlation analyses of these endpoints, there exists the potential to identify markers or patterns that hold significance for subsequent studies. The insights yielded by this research are poised to enrich our comprehension of dual use patterns and consequently, provide valuable insights that can be harnessed in devising tobacco harm reduction strategies grounded in empirical evidence.

## 2 Methods

The study is designed to assess the impact of different well-defined dual-use patterns in term of degree of harm reduction that can be achieved when smokers are curtailing their CC consumption by switching to EC use. The combination of biomarkers signaling exposure and potential harm, cardiovascular and respiratory functionalities, and self-reported outcomes by study participants will be used to characterize the extent of harm reduction as a consequence of reducing exposure to cigarette smoke toxicants. It is anticipated that the study population of dual users can be assigned to three sub-groups: “strong switchers” (>80% reduction in CPD from baseline); “moderate switchers” (50–80% reduction in CPD from baseline), “poor switchers” (<50% reduction in CPD from baseline).

### 2.1 Study population

A total of 300 adult smokers will be recruited for the study from the clinical study site subject pool and public advertisement. Advertisements will include social, digital and print media to publicize the trial to local communities in the catchment area to reach out potential participants. Inclusion/exclusion criteria will be included in the publicity material. To confirm their smoking status, an exhaled Carbon Monoxide (eCO) measurement will be taken at the Screening Visit. The cut-off for confirmation as a smoker will be an exhaled CO level ≥ 7 parts per million (ppm). Each participant identified as a smoker will be offered access to a smoking cessation program aimed at helping participants quit smoking. Those who decline being referred to the smoking cessation program will be eligible.

Eligible subjects will be stratified into two study groups: one consisting of 250 smokers who express intention to reduce CC consumption by switching to a vaping product of their choice (Study Group A); and the other consisting of a reference group of 50 sex- age-matched smokers who are not interested to quit or reduce CC consumption (Study Group B).

Participants will be required to satisfy all of the following criteria both at screening (day −7) and at Visit 1 (day 0):

#### 2.1.1 Inclusion criteria

- ≥ 19 years of age
- *Solus* smokers of combustible cigarettes (≥ 15 cigarettes/day)
- History of regular smoking for at least 12 consecutive months
- Verified smoking status (eCO ≥ 7 ppm)
- Willingness to switch to a vaping product and to try reducing combustible cigarette consumption (Study Group A only)
- Refusal to participate in smoking cessation programs
- Physically and mentally healthy, as judged by the principal investigator based on medical history, vital signs (blood pressure, pulse rate) and spirometry
- Given written informed consent to participate in the study

#### 2.1.2 Exclusion criteria

- Intention to quit smoking within the next 30 days
- Known clinically significant cardiovascular, respiratory, psychiatric, or other major disorder that, in the opinion of the principal investigator, would jeopardize the safety of the participant or impact on the validity of the study results
- Regular use of any medication
- A significant history of alcohol or drug abuse, as judged by the principal investigator
- Use of any nicotine (e.g., e-cigarettes, nicotine pouches) or tobacco product (e.g., heated tobacco products - HTPs, oral smokeless) other than their own combustible cigarettes within 3 months of screening
- Use of nicotine replacement therapy or other smoking cessation therapies within 3 months of screening
- Pregnant or breast feeding or intention to become pregnant during the course of the study
- Active participation in another clinical trial

### 2.2 Study objectives and endpoints

#### 2.2.1 Primary objective

This study aims to investigate the impact of substituting combustible cigarettes (CC) with potentially less harmful nicotine delivery products (specifically vaping devices), in relation to the overall extent of cigarette substitution.

By evaluating the extent of the substitution (which is assessed by self-reported consumption and objective biomarkers), the research aims to quantify the potential risk of dual using both combustible cigarettes and vaping devices.

The assessment will include established Biomarkers of Exposure (BoEs) indicative of self-reported CC substitution, along with evaluations of cardio-respiratory endpoints. The study will also explore the correlation between usage band behavior and BoEs representing harmful components. Collectively, these endpoints will enable a scientific assessment of different profiles in dual users and associated risks.

##### 2.2.1.1 Primary endpoints

1. Extent of CC Smoking Substitution with vaping products over time (30–180 days): The extent of reduction in CC smoking consumption and of concurrent increase in the use of vaping products will be quantified using self-reported consumption protocols verified by suitable biomarkers of exposure. This approach ensures a comprehensive characterization of the participants’ shift in smoking behavior.
2. Biomarkers of Exposure: the selected biomarkers have been chosen to gauge participants’ exposure to a range of harmful and potentially harmful substances. These biomarkers provide objective data on the presence of specific components, allowing for a more accurate evaluation of potential health risks associated with both combustible cigarettes and vaping products by correlating BoE levels with the use pattern (cigarettes per day; vape liquid per day). The panel of BoE includes:
  - Acrolein (3-HPMA)
  - 1,3-Butadiene (MHBMA)
  - Propylene Oxide (2-HPMA)
  - Crotonaldehyde (HMPMA)
  - Benzene (SPMA)
  - Styrene (PHEMA)
  - Glycidol (DHPMA)
  - Isoprene (IPMA)
  - Toluene (SBMA)
  - Ethylene Oxide (HEMA)
  - Acrylonitrile (CEMA/CeVal)
  - Acrylamide (AAMA/GAMA/GlyVal)
  - Metabolites of polyaromatic hydrocarbons (benzo[a]pyrene, pyrene, phenanthrene, naphthalene)
  - Aromatic amines (3−/4-aminobiphenyl, 2-aminonaphthalene, ortho-toluidine)
  - total nicotine equivalents
  - (Methylnitrosamino)-1-(3-pyridyl)-1-butanol (NNAL)
  - N-nitrosonornicotine (NNN)
  - Propylene glycol

In summary, this study seeks to provide valuable insights into the dynamics of dual use of combustible cigarettes and vaping products. By employing a combination of self-reported data, biomarkers, and exposure assessments, the research aims to offer a comprehensive understanding of the substitution process’s impact on participants’ smoking behaviors and toxicant exposure and eventually potential harm.

#### 2.2.2 Secondary objectives

The secondary objectives encompass the determination of BoEs as well as non-targeted screening of the urinary exposome, breathome and adductome. In addition, the assessments of quality of life and BoPH may be indicative of the cardio-respiratory health of the participants after (partly) switching. Moreover, correlation analyses of the use behavior with exposure via BoE and BoPH in urine, plasma and EB in compliant subjects (biochemically verified with biomarkers of compliance) will be performed.

##### 2.2.2.1 Secondary endpoints

- Biomarkers of potential harm:
  - Eicosanoids in urine,
  - soluble intercellular adhesion molecule 1 (sICAM-1) in plasma,
  - growth differentiation factor 15 (GDF-15) in plasma.
- Cardio-respiratory endpoints:
  - VO2 max/Chester step test,
  - Spirometry,
  - Respiratory Symptoms Questionnaire (RSQ).
- Non-targeted methods for the screening of the urinary exposome, the hemoglobin adductome and the breathome (exhaled breath - EB)
- Correlation of use behavior with exposure via BoE and BoPH in urine, plasma and EB in compliant subjects (biochemically verified with biomarkers of compliance)

##### 2.2.2.2 Normalization

- Urinary creatinine for normalization of urinary biomarker concentrations

### 2.3 Study design

**Figure 1:**
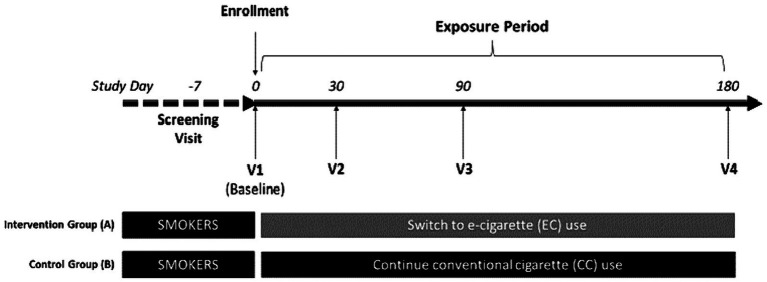
Study design of MAGNIFICAT.

This is a 6-month prospective, single center, clinical trial with two parallel study groups: one consisting of 250 smokers who express interest to reduce CC consumption by switching to a vaping product of their choice (Study Group A); and the other consisting of a reference group of 50 sex- age-matched smokers who are not interested to quit or reduce CC consumption (Study Group B) (Figure 1). The setting for the study will be an ambulatory (outpatient) setting. The design of the trial follows the rules set by the Standard Protocol Items: Recommendations for Interventional Trials (SPIRIT) guidelines (Appendix, SPIRIT checklist).

#### 2.3.1 Study visits

Participants will attend a total of five clinic visits (Figure 1):

- Day −7 to Day −1: Screening Visit
- Day 0: Baseline Visit, Visit 1
- Day 30: Visit 2
- Day 90: Visit 3
- Day 180: Visit 4

Potential participants will attend a screening visit within 7 days prior to baseline visit (visit 1) to check their eligibility criteria (Figure 1; Tables 1, 2). At screening, socio-demographic data, detailed medical history (including medication use), and detailed history of smoking, e-cigarette, heated tobacco products (HTPs) and oral nicotine/tobacco use will be collected. Self-reported smoking status will be objectively verified by measuring exhaled breath carbon monoxide (eCO) levels. Additionally, participants’ intention to quit smoking will be assessed. All patients will be offered a smoking cessation program according to the local guidelines. Any participant who expresses the intention of booking for the cessation program or to quit smoking in the next 30 days will be urged to do so and not be recruited in the study. Patients taking part in the study will be informed that they are free to quit smoking and withdraw from the study at any time. Moreover, participants will be encouraged to quit smoking at every contact timepoint throughout the whole study. Participants’ intention to try and switch to EC use will be assessed; those who are not interested to try and switch to EC use can be offered to enroll only in the cigarette smoking control group of the study if they wish to (Study Group B). Before discharging subjects from the clinic, subjects will be invited to attend the baseline visit (visit 1), given a sterile,

screw-top urine container and instructed on how to collect and store their morning urine sample just before bringing it to the clinic on the day of their Baseline visit.

**Table 1:** Study schedule of procedures (intervention group; EC group).

Procedure	Screening visit	Visit 1	Visit 2	Visit 3	Visit 4
	Day − 7	Day 0	Day 30	Day 90	Day 180
Informed consent		X					
Inclusion/exclusion criteria	X	X						
Demographics^1^	X							
Medical/Surgical history	X								
Smoking history	X								
Cigarette consumption	X	X	X	X	X
Exhaled carbon monoxide	X	X	X	X	X
FTCD		X					
Weight, height, and BMI			X	X	X	X
Blood pressure, pulse rate			X	X	X	X
Interest to try ECs (Y/N)^2^	X						
EC familiarization		X						
EC use checks				X	X	X
Tracker APP installation			X					
Tracker App Training			X						
Lung function/spirometry			X	X	X	X
Chester Step test (VO2max)			X	X	X	X
Questionnaires: RSES			X	X	X	X
eDiary Evaluation/eCRF			X	X	X	X
Provision of urine collection kit	X	X	X	X	
Collection of biospecimens (urine, blood, exhaled breath)		X	X	X	X
Adverse events reporting/e-CRF			X	X	X	X
Provision of vaping products (switching arm only)			X^3^	X^3^	X^3^	

^1^Demographics include sex, age, ethnicity, and race.

^2^If answer is “NO,” subject can enroll only in the cigarette smoking control group of the study.

^3^For detailed supply schedule see Table 2.

**Table 2:** Study schedule of procedures (control group; CC group).

Procedure	Screening visit	Visit 1	Visit 2	Visit 3	Visit 4
	Day − 7	Day 0	Day 30	Day 90	Day 180
Informed consent		X					
Inclusion/exclusion criteria	X	X						
Demographics^1^	X							
Medical/Surgical History	X								
Smoking History	X								
Cigarette consumption	X	X	X	X	X
Exhaled carbon monoxide	X	X	X	X	X
FTCD		X					
Weight, height, and BMI			X	X	X	X
Blood pressure, pulse rate			X	X	X	X
Tracker APP Installation			X					
Tracker App Training			X						
Lung function / spirometry			X	X	X	X
Chester Step test (VO2max)			X	X	X	X
Questionnaires: RSES			X	X	X	X
eDiary Evaluation / eCRF			X	X	X	X
Provision of urine collection kit	X	X	X	X	
Collection of biospecimens (urine, blood, exhaled breath)		X	X	X	X
Adverse events reporting / e-CRF			X	X	X	X

^1^Demographics include sex, age, ethnicity, and race.

Within 7 days of the screening visit, eligible participants will attend the baseline visit (visit 1). Eligibility criteria will be verified again. Self-reported smoking status will be objectively verified by measuring exhaled breath carbon monoxide (eCO) levels. Smokers will be reminded of the risks associated with smoking prior to enrolment onto the study and that they are free to voluntarily quit smoking/nicotine and/or withdraw from the study at any time.

Smokers intending to try and switch to EC use will be assigned to study group A (EC switching group); those not interested will be assigned to study group B (cigarette smoking control group). Subjects in group A will receive one vaping kit and will be asked to trial and familiarize with vaping products provided by the study investigator so that can choose the product of their preference to try and reduce smoking as much as they can.

The extent of reduction in CC consumption will be self-reported (and tracked by an App tracker) and used to characterize dual usage into “Poor dual-use” (1–49% CPD reduction from baseline), “Moderate dual-use” (50–79% CPD reduction from baseline) and “Intensive dual-use” | (80–99% CPD reduction from baseline). Subjects in group B will continue to use their usual brand of tobacco cigarette.

All participants will be undergoing a series of baseline measurements (as outlined in Tables 1, 2), including: (1) resting heart rate (HR), systolic (SBP) and diastolic (DBP) blood pressure; (2) body weight, height and Body Mass Index (BMI); (3) Fagerstrom test for cigarette dependence (FTCD), a 6-item questionnaire used to measure the intensity of physical dependence related to cigarette smoking; (4) Respiratory Symptom Experience Scale (RSES), a 5-item questionnaire designed to measure the frequency and severity of respiratory symptoms; (5) spirometry; and (6) Chester step test to determine maximal aerobic capacity (i.e., V̇O_2_ max).

We will collect, process, and store blood samples (including plasma, and washed erythrocytes), as well as samples of exhaled breath (EB). Additionally, containers with participants’ morning urine sample will be handed over to the principal investigator and stored. Samples will be shipped for biomarkers analyses to a centralized lab.

Participants’ smartphones will be equipped with a dedicated tracker application. This application is designed to monitor individual tobacco cigarette consumption and EC usage throughout the study. The tracker application will also identify any protocol violations, collect adverse events and send reminders (e.g., next scheduled appointment, study restrictions, instructions) throughout the study duration. Subjects will receive training and instructions on how to use the app. The use of a dedicated tracker application adds an innovative element to continuously collect data and enhances adherence to the study protocol.

Prior to check-out, subjects will receive one vaping kit together with a 15-day supply of pre-filled. e-liquid pods of their choice (as outlined in Table 3), and a new sterile, screw-top urine container to collect and store their morning urine sample for the next clinic appointment (visit 2).

**Table 3:** Study schedule of EC supply and use checks (only for EC switching group).

Procedure	Screening	Day 0 (V1) BL	Day 15	Day 30 (V2)	Day 60	Day 90 (V3)	Day 120	Day 150	Day 180 (V4)
EC use (Y/N)			X	X	X	X	X	X	X
Provide EC Device^*^			X													
Hand out 15-day supply of pods^**^			X	X											
Hand out 30-day supply of pods^***^						X	X	X	X	X	
Product use checks (collect used and unused pods)				X	X	X	X	X	X	X

BL = baseline; V = visit; C-F NA = combustion-free nicotine alternative.

^*^Participants will receive one vaping kit.

^**^Participants will receive 15 e-liquid pods of their choice (average consumption estimated: one pod/day).

^***^Participants will receive 30 e-liquid pods of their choice (average consumption estimated: one pod/day).

Following the initial baseline visit (Visit 1), participants will be invited to attend three subsequent clinical appointments at 30 (visit 2), 90 (visit 3), and 180 days (visit 4). The same measurements and sample collections conducted during the baseline assessment will be repeated in these consecutive follow-up visits (as outlined in Tables 1, 2). This approach aims to track modifications in biomarkers of exposure and biological responses, alongside improvements in health indicators in the context of transitioning from tobacco smoking to adopting ECs. The monitoring will persist over a continuous span of 6 months, enabling a thorough and consistent evaluation of the observed changes.

Throughout the study, participants in the EC switching group (Group A) will be provided with an appropriate number of prefilled e-liquid pods at clinical and additional non-clinical visits between the clinical visits (as outlined in Table 3). These non-clinical visits are designed to supply consumables and provide an opportunity for study investigators to stimulate retention and check compliance.

Throughout the study, tobacco cigarette consumption, EC usage, and adverse events will be regularly monitored with the tracker APP and noted in the personal diaries in order to capture the extent of CC smoking along with the magnitude of substitution at each study visit. Monitoring of cigarette consumption and daily EC usage will be also achieved by asking participants in the switching group (Group A) to return all empty, part-used, and unused prefilled e-liquid pods from the previous study period at each visit (as outlined in Table 3). The use of a dedicated tracker application adds an innovative element to continuously collect data and enhances adherence to the study protocol.

Prior to check-out, subjects will receive urine containers to collect and store their morning urine sample for the next clinic appointment and sufficient supplies of pre-filled e-liquid pods until their next visit (as outlined in Table 3). At the final visit (visit 4), no more pods will be dispensed.

### 2.4 Statistical and analytical plans

Complete and specific details of the statistical analyses will be described and fully documented in the Statistical Analyses Plan (SAP). The SAP will be finalized prior to database lock.

### 2.5 Sample size estimation

No previous studies have evaluated the impact of EC dual use on BoE, so the sample size was based on a series of assumptions, informed by prior experience in a previous study that evaluated BoE in users of four different CC alternatives (32–34). In this study, significant between-groups differences were demonstrated using a sample size of 10 participants group. However, this study was carried out over a short period and involved protocol-mandated diet and lifestyle controls. As MAGNIFICAT is a long term, real world study, a series of generous uplifts to the base case of 10 patients per group were made for the purposes of our sample size estimate:

- To allow for unequal distribution of patients across the dual-use categories at baseline, the sample size was increased by 40%,
- In order to compensate for reduced between-groups differences in biomarkers and lack of lifestyle control, the sample size was doubled,
- To allow for migration of patients across groups over the course of the study and to maximize the chances of having sufficient residual power at the 6-month evaluation point, the sample size was then further increased by 50%,
- Finally, in order to mitigate withdrawals and dropouts over the course of 6 months follow-up, the sample size was finally increased by an additional 20%.

The final sample size for analyses based on these assumptions is 42 participants per group. After uplift for withdrawals and dropouts, this yields an estimated total of 252 participants to be recruited, with an additional 50 participants will be recruited for an external control group who have chosen to continue smoking and who will not be offered ECs.

### 2.6 Definition of dual-use categories

For each evaluation point (1 month, 3 months, 6 months), information on CPD for the 30 days preceding will be extracted from the CRF. Mean CPD over the evaluation period will be calculated and then used to assign dual-use category membership:

- “Smoker”: No change or increased CC consumption from baseline
- “Poor dual-use”: 1–49% CPD reduction from baseline
- “Moderate dual-use”: 50–79% CPD reduction from baseline
- “Intensive dual-use”: 80–99% CPD reduction from baseline
- “Quitter”: 100% CPD reduction from baseline

### 2.7 Analyses populations

#### 2.7.1 Full analyses (FA) Population

All subjects who received ECs, who participated in at least one post-baseline assessment.

#### 2.7.2 Per protocol (PP) population

All subjects in the full analyses population who participated in assessment at 1 month, 3 months and 6 months and did not have any significant protocol deviations, defined as:

- Failure to attend for one or more assessment visits within the agreed time windows
- More than 5 missing CPD diary entries in the 30 days preceding an assessment visit
- Any missing samples available (blood, urine, exhaled breath) at any of the assessment visits
- Use of nicotine replacement or non-study vaping products

A modified PP population was defined as all subjects in the per protocol population who were also placed in the same dual-usage category at all three post-baseline assessments.

#### 2.7.3 Safety population

All subjects who received any study treatment (including control) but excluding subjects who dropped out prior to receiving any treatment.

### 2.8 Statistical analyses for efficacy

#### 2.8.1 Primary analyses

The primary analyses will be carried out on the FA population based on the primary outcome: change from baseline in urinary CEMA. The null hypothesis to be tested is that the mean values for urinary CEMA for each of the dual-use categories is derived from the same population. The hypothesis will be tested independently at 1 month, 3 months, and 6 months, with the dual-use categories being re-assigned, where necessary, at each time point. These analyses will be also be repeated on the external smoking control group.

In order to allow for non-normality in biomarker distribution, the analyses will be carried out using the Jonckheere–Terpstra trend test. In the event that the null hypothesis is rejected, pairwise comparisons will subsequently be carried out using the approach of Conover (35).

#### 2.8.2 Secondary analyses

##### 2.8.2.1 Secondary analyses of primary endpoint

The primary analyses will be repeated using the PP population.

##### 2.8.2.2 Analyses of secondary endpoints

The same analytical approach as described for the primary analyses will be applied to each of the three secondary BoE endpoints: whole blood CeVal, urinary NNAL and urinary PG at 1 month, 3 months and 6 months. The analyses will be carried out using the FA population for the intervention groups and will also be carried out on the external smoking control group.

#### 2.8.3 Exploratory analyses

##### 2.8.3.1 Analyses of alternative BoE

Results for a wide range of other potential BoE, listed below will be pre-screened for their potential to serve as a useful metric in dual-users. Where ordered numerical differences between dual-use categories as noted to exist, the specific BoE will be analysed using the same analytical approach as outlined for the primary outcome. The analyses will be carried out using the FA population for the intervention groups and will also be carried out on the external smoking control group.

##### 2.8.3.2 Analyses of BoPH

For these analyses, the dual-use categories will be assessed based on long term CC usage. The statistical analyses will be carried out using the analytical approach outlined for the primary analyses. This analyses will be carried out on both the PP and modified PP populations.

### 2.9 Statistical analyses for safety

Safety analyses will be carried out on the Safety Population. A summary table will present the number of adverse events (AE) broken down by severity, treatment-emergent adverse events (TEAE) and serious adverse events (SAEs).

AEs will be coded using MedDRA and assigned grades based on NCI CTCAE, Version 4.03. The number and percentage of subjects reporting TEAEs will be tabulated by the worst CTCAE grade, system organ class, and preferred term, with a breakdown by treatment group. Similarly, the number and percentage of subjects reporting treatment-emergent SAEs will be tabulated, as well as treatment-emergent AEs/SAEs considered related to study treatment and TEAEs leading to discontinuation of study treatment, respectively.

A by-subject AE (including treatment-emergent) data listing including, but not limited to, verbatim term, preferred term, system organ class, CTCAE grade, and relationship to study treatment will be provided. Deaths, other SAEs, and other significant AEs, including those leading to permanent discontinuation from study treatment of a subject, will be listed.

## 3 Results

Subject recruitment will start in Feb 2024 and enrolment is expected to be completed by Aug 2024. Results will be reported early in 2025.

## 4 Discussion

The argument against the use of ECs often leverages the concept of dual use, suggesting an absence of risk/harm reduction. This has deterred many smokers from adopting these products to curtail their smoking habit. Therefore, a clear understanding of the risks associated with combining conventional cigarettes (CC) and EC use is crucial. Dual users vary significantly in their usage patterns, creating distinct impact levels on toxin exposure and smoking-related disease risks. By compiling a robust dataset on well-defined stratification of dual-use patterns, this study will serve as a foundational resource, offering a deeper understanding of the health implications of dual use. The methods and processes specified in this protocol are all aimed at better understanding the risks associated with dual use and at ensuring that the public and policy makers receive correct and reliable evidence-based information about dual use. The MAGNIFICAT study will stand out as the first clinical trial being adequately powered to collect such evidence. Ultimately, this study seeks to dispel misperceptions surrounding dual use and pave the way for informed decisions regarding the use of nicotine/tobacco products.

This study exhibits several notable strengths: (1) the sample size robust enough to facilitate the categorization of dual users into various sub-groups based on their distinctive usage patterns; (2) it utilizes highly specific and sensitive biomarkers to objectively verify smoking reduction and vaping use, enhancing the accuracy of compliance assessment and avoid relying solely on self-reporting; (3) a wide array of biomarkers, including health effect indicators, is thoroughly investigated, providing comprehensive insights; (4) the non-targeted analyses in urine, blood, and exhaled breath may allow a comprehensive understanding of the absolute exposure in dual users which is important to better understand the risk related to dual use; (5) notably, the study incorporates a meticulously designed statistical analyses plan, ensuring methodical and accurate data interpretation; (6) the meticulous monitoring of cigarette consumption and/or e-cigarette use throughout the study is facilitated by a specialized tracker app technology. This technology enables a detailed characterization of dual usage patterns. Furthermore, participants will be requested to return all empty, partially used, and unused consumables, bolstering the completeness of data collection: and (7) it harnesses a collaborative effort between two leading entities in the field of nicotine/tobacco research: ECLAT srl, a spin-off company from the University of Catania with a proven track record in executing high-quality switching trials, and ABF GmbH, an independent research organization specializing in biomarker analyses and exposure assessment.

While the study boasts several strengths, it is important to acknowledge its limitations.

Firstly, maintaining a sufficient number of dual users within each sub-group might pose challenges in ensuring robust statistical power. However, the sample size is deliberately designed to ensure a minimum of 20 subjects per sub-group, which should generally enable the detection of significant changes. In the unlikely scenario of a shortfall in subjects, supplementing the study population with additional participants could be considered. Secondly, although the six-month study duration is anticipated to adequately reveal alterations in toxicant exposure resulting from concurrent combustible cigarette (CC) smoking and vaping initiation, certain biomarkers of potential harm and health indicators may exhibit slow response rates, potentially rendering their changes undetectable. Thirdly, the exploratory analyses of biomarkers of potential harm (BoPH) might encounter a longer lag time between changes in exposure to ECs and corresponding alterations in markers compared to biomarkers of exposure (BoE). To ensure robust conclusions, with regard to BoPH, it is advisable to confine the analyses to individuals whose dual-use category remains consistent throughout the entire six-month follow-up. If a substantial number of participants shift between dual-use categories during the study, there is a risk that the BoPH analyses might lack adequate power.

It is planned to publish the study results in reputable, peer-reviewed, open access, scientific journals. Besides the publications, the main outcomes shall be presented at scientific conferences and published in a suitably adapted version in a popular scientific journal or as a whitepaper to be sent to policy makers, media and stakeholders. We will also post our findings on the University website (Center of Excellence for the acceleration of harm reduction – CoEHAR at the University of Catania), and related social media accounts (e.g., Linkedin, Twitter, Instagram, YouTube) and in the CoEHAR monthly newsletter. Dedicated press releases will be also prepared and sent to national and international media outlets. Video interviews and podcasts are planned to be realized to disseminate results.

The dissemination strategy for our study results involves publication in reputable, peer-reviewed, open-access scientific journals. Additionally, the primary outcomes will be presented at scientific conferences and adapted suitably as a whitepaper aimed at policy makers, media representatives, and stakeholders. To ensure broader accessibility, our findings will be shared on the University website, specifically through the Center of Excellence for the acceleration of harm reduction (CoEHAR) at the University of Catania, as well as across various social media platforms such as LinkedIn, Twitter, Instagram, YouTube, and in the CoEHAR monthly newsletter. Moreover, we plan to create dedicated press releases to disseminate the findings to national and international media outlets. To further engage with audiences, we aim to conduct video interviews and podcasts to share and discuss our results.

## Author contributions

RP: Conceptualization, Investigation, Methodology, Supervision, Validation, Writing – original draft, Writing – review & editing. NP: Conceptualization, Investigation, Methodology, Writing – original draft, Writing – review & editing. MS: Investigation, Methodology, Writing – original draft, Writing – review & editing. JB: Conceptualization, Methodology, Visualization, Writing – original draft, Writing – review & editing. CR: Conceptualization, Methodology, Writing – review & editing. PC: Conceptualization, Formal analysis, Methodology, Visualization, Writing – review & editing. JW: Formal analysis, Project administration, Validation, Writing – original draft, Writing – review & editing. DC: Conceptualization, Methodology, Supervision, Visualization, Writing – review & editing.
